# Clinical Characteristics, Outcomes, and Risk Factors for Patients with Diffuse Large B-Cell Lymphoma and Development of Nomogram to Identify High-Risk Patients

**DOI:** 10.1155/2022/8395246

**Published:** 2022-11-17

**Authors:** Jinrong Zhao, Yan Zhang, Wei Wang, Wei Zhang, Dao-bin Zhou

**Affiliations:** Department of Hematology, Peking Union Medical College Hospital, Peking Union Medical College, Chinese Academy of Medical Sciences, Beijing, China

## Abstract

**Objectives:**

To analyse the clinical features, outcomes, and risk factors of patients with diffuse large B-cell lymphoma (DLBCL) in China, with the aim to establish a new prognostic model based on risk factors.

**Methods:**

Clinical features and outcomes of 564 patients newly diagnosed with DLBCL from Jan 2009 to May 2017 were analyzed retrospectively. Variables were screened by LASSO regression and nomogram was constructed.

**Results:**

The 5-year overall survival (OS) of the cohort was 75%. The 5-year OS of patients differentiated by International Prognostic Index (IPI) score was 90% (score 0–2), 73% (score 3), and 51% (score 4-5), respectively. Age > 60, Eastern Cooperative Oncology Group (ECOG) > 1, Ann Arbor stage III-IV, bone marrow involvement, low level of albumin (ALB), and lymphatic/monocyte ratio (LMR) were independent predictors of OS. The predictive model was developed based on factors including age, bone marrow involvement, LMR, ALB, and ECOG scores. The predictive ability of the model (AUC, 0.77) was better than that of IPI (AUC, 0.74) and NCCN-IPI (AUC, 0.69). The 5-year OS of patients in the low-, intermediate-, and high-risk groups identified by the new predictive model was 89%, 70%, and 33%, respectively.

**Conclusions:**

The new prediction model had better predictive performance and could better identify high-risk patients.

## 1. Introduction

Diffuse large B-cell lymphoma (DLBCL) is the most common histological subtype of non-Hodgkin lymphoma (NHL), accounting for approximately 25% of NHL cases [1]. The disease is aggressive and requires aggressive medical intervention after diagnosis. The International Prognostic Index (IPI) [2] has played an important role in determining the prognosis of patients with DLBCL over the past two decades. With the addition of rituximab to the CHOP or CHOP-like regimen, the prognosis of patients in each risk group according to IPI improved. New prognostic scoring systems, such as R-IPI [3] and NCCN-IPI [4], have emerged to better discriminate the survival of patients with DLBCL.

With the innovation of immunohistochemistry and molecular examination techniques and the optimization of treatment strategies, we need more accurate prognostic models to identify very high-risk DLBCL patients or biological heterogeneity to guide individualized treatment. The application of Lasso regression [5] facilitated the selection of variables, and the use of nomograms [6] proved to be of better predictive value. In this study, we analyzed the clinical characteristics of patients with DLBCL and explored the factors influencing survival, screened variables through Lasso regression, and constructed a nomogram to stratify the prognosis of patients with DLBCL.

## 2. Patients and Methods

### 2.1. Patients

From Jan 2009 to May 2017, a total of 564 patients newly diagnosed with DLBCL according to WHO classification [7] by three specialized pathologists were included. Patients who did not have complete clinical and immunohistochemical data or who were diagnosed but not treated in our hospital were excluded (*n* = 65). Because primary central nervous system lymphomas are highly heterogeneous entities, we also exclude them from our study (*n* = 41). Baseline data were collected such as gender, age, B symptoms, performance status, number of extranodal involvement, presence of bulky disease (≥7.5 cm), Ann Arbor stage, cell of origin, lactate dehydrogenase (LDH), albumin (Alb), white blood cell count (WBC), hemoglobin (HGB), platelet (PLT), absolute lymphocyte count (ALC), absolute monocyte count (AMC), the ratio of lymphocyte to monocyte (LMR), and D-dimer. All patients in the cohort were routinely evaluated by lumbar examination before treatment. Bone marrow examination was performed before treatment to determine whether there was bone marrow involvement, and the efficacy was assessed by whole-body computed tomography scan (CT) or positron emission tomography/computed tomography (PET-CT).

### 2.2. Treatment, Follow-Up, and Outcome

The first-line therapy for DLBCL patients was the R-CHOP or R-CHOP-like regimen [8]. Addition of intravenous methotrexate (1 g/m^2^, four times) [9] injection was used as central prophylaxis in patients at central high risk. For elderly patients >70 years of age, we divided them into three groups: fit, unfit, and frail according to the comprehensive geriatric assessment (CGA) [10], and they were treated with R-CHOP, R-mini-CHOP [11], and R2 (rituximab 375 mg/m^2^ on d1, lenalidomide 25 mg on day 1–14) regimens, respectively. Follow-up was conducted by making phone calls, consulting medical records, or the electronic follow-up system we run. 61 (10.8%) patients were lost to follow-up until the final follow-up period of May 1, 2022. Overall survival (OS) was calculated from the time of diagnosis to death for any cause or the last follow-up.

### 2.3. Model Foundation and Validation

66.7% (*n* = 376) of the original dataset was randomly selected as a training cohort and the rest (*n* = 188) as a validation cohort. Univariate and multivariate Cox regression analyses were performed to screen potential variables associated with OS. The selected significant variables (*P* value <0.5) were then used in the least absolute shrinkage and selection operator (LASSO) regression algorithm, and then, a predictive nomogram was constructed. The area under receiver operating curve (AUC) is used to evaluate the performance of the model. According to the analysis results, calibration curves are drawn to determine whether the predicted and actual survival probabilities are consistent. The total score for each patient was assessed using nomogram in an externally validated cohort and used as an independent factor for Cox regression analysis.

### 2.4. Statistical Analysis

The survival of patients was analyzed by Kaplan–Meier survival curve, and differences between groups were compared by the log-rank test. Graph Pad Prisma 9.0 and R statistical software 4.1.3 (https://www.r-project.org/) were used to perform the statistical analyses. *P* value <0.05 was considered to be statistically different.

## 3. Results

### 3.1. Clinical Characteristics

**Figure 1:**
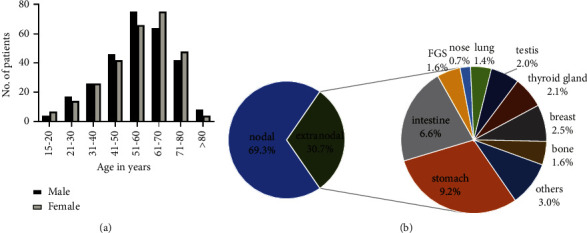
Distribution of age (a) and primary extranodal sites (b) in diffuse large B-cell lymphoma. CNS, central nervous system; FGS, female genital system.

The baseline clinical characteristics of the entire cohort are shown in Table 1. 282 (50.0%) patients included in this study were male. The median age of all patients was 58 years (range: 15–90). The age distribution at diagnosis is shown in Figure 1(a). More patients were non-GCB subtype (50.7%) and advanced stage (72.0%). Fewer patients had bulky disease (13.7%), bone marrow (12.6%), or CNS involvement (6.4%). The gastrointestinal tract (GIT) constituted the most common site of primary extranodal DLBCL, accounting for 15.8% (89/564) of DLBCL cases (9.2% stomach and 6.6% intestine). Patients with primary breast involvement rank second (14, 2.5%), followed by the thyroid gland (12, 2.1%), testis (11, 2.0%), female genital system (9, 1.6%), bone (9, 1.6%), and others.  Figure 1(b) shows the site distribution of extranodal lymphomas. No significant difference in clinical characteristics was found between the training and validation cohorts (*P* > 0.05).

### 3.2. Outcome

**Figure 2:**
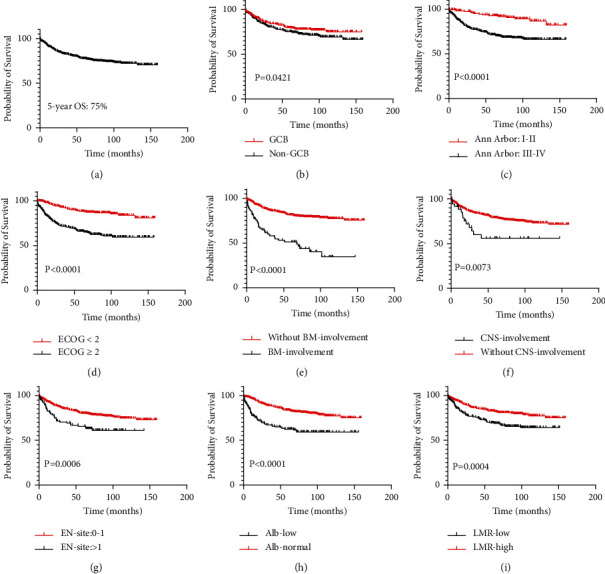
Clinical outcomes for diffuse large B-cell lymphoma. Overall survival of the complete cohort (a) and in each subgroup according to pathological classification (b), Ann Arbor stage (c), ECOG performance status (d), bone marrow involvement (e), CNS involvement (f), extranodal site involvement (g), albumin levels (h), and LMR levels (i). CNS, central nervous system; LMR, lymphocyte-monocyte ratio; BM, bone marrow; EN-site, extranodal site. Statistical significance was tested with log-rank. *P* < 0.05 means a significant difference.

At the last follow-up, 18.6% (105/564) of patients were lost to follow-up, 22.5% (127/564) of patients were dead. The 5-year OS of patients with DLBCL was 75% (Figure 2(a)). Patients with GCB subtype (Figure 1(b)), Ann Arbor stage I-II (Figure 2(c)), ECOG score 0-1 (Figure 2(d)), and fewer extranodal involvement sites (Figure 2(g)) had better clinical outcome. The overall survival of patients with bone marrow (Figure 2(e)) or CNS (Figure 2(f)) involvement was significantly lower than those without. Patients with lower Alb or LMR levels resulted in lower 5-year OS rate (59% vs. 82%, *P* < 0.0001; 67% vs. 81%, *P*=0.0004) (Figures 2(h) and 2(i)).

### 3.3. Risk Factors

The univariate and multivariate analyses results for patients with DLBCL are presented in Table 2. On the basis of the univariate results, gender, age, ECOG, B symptom, Ann Arbor stage, IPI score, cell of origin, BM involvement, CNS involvement, extranodal site, LDH level, LMR, and ALB were significantly associated with survival. However, in the multivariate analysis, age > 60 (HR: 2.086, 95% CI: 1.371–3.175), ECOG > 1 (HR: 2.666, 95% CI: 1.790–3.970), Ann Arbor stage III-IV (HR: 1.857, 95% CI: 1.035–3.333), BM involvement (HR: 2.024, 95% CI: 1.413–2.898), low LMR (HR: 1.605, 95% CI: 1.128–2.283), and low Alb (HR: 1.548, 95% CI: 1.088–2.202) were independent risk factors of OS in patients with DLBCL.

### 3.4. Parameter Selection

**Figure 3:**
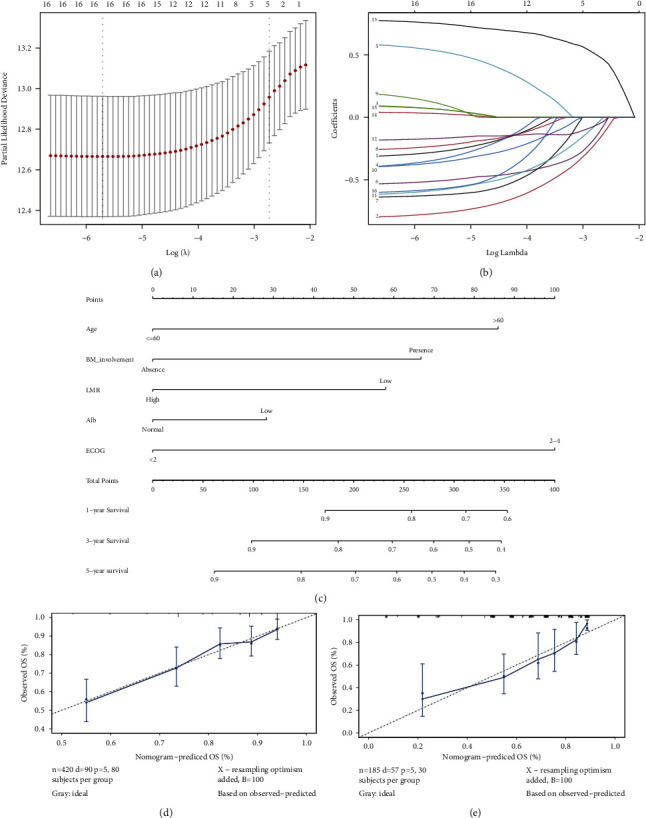
Construction and validation of a predictive nomogram. Lasso Cox regression performed to identify the factors closely related to the prognosis of DLBCL (a-b). The nomogram for predicting the OS of patients with DLBCL at 1, 3, and 5 years (c). Each patient's 5-factor score (i.e., age >60 years, 86 points) can be derived from the nomogram, and the sum of the above scores is added to the individual's total score. The estimated probability of occurrence of this total score was the overall survival of the patient. Calibration curve for predicting OS at 5 years in the training (d) and the validation cohort (e).

A total of 16 candidate parameters (age, gender, cell of origin, Ki-67, bulky disease, BM involvement, CNS involvement, B symptom, LDH, D-dimer, Alb, LMR, ECOG, Ann Arbor stage, extranodal site, and HBsAg) in the training cohort were screened and verified using Lasso Cox regression (Figures 3(a) and 3(b)). Finally, several factors, age, BM involvement, LMR, Alb, and ECOG performance status, were independently associated with the prognosis of patients with DLBCL and were included in subsequent nomogram.

### 3.5. Construction and Validation of the Predictive Nomogram

The predictive model (Figure 3(c)) was constructed by 5 factors identified from the results of Lasso regression. In this model, ECOG performance status ≥2 was assigned the highest score of 100, age > 60 y, bone marrow involvement, low levels of LMR, and low Alb was scored 86, 67, 58, and 28 points, respectively. The AUC for the nomogram was 0.77 (95% CI: 0.70–0.82), and the calibration curves of the nomogram showed great consistency between the predicted OS rates and actual observations outcome (Figures 3(d) and 3(e)).

### 3.6. Comparison with Current Prognostic Scoring Systems

**Figure 4:**
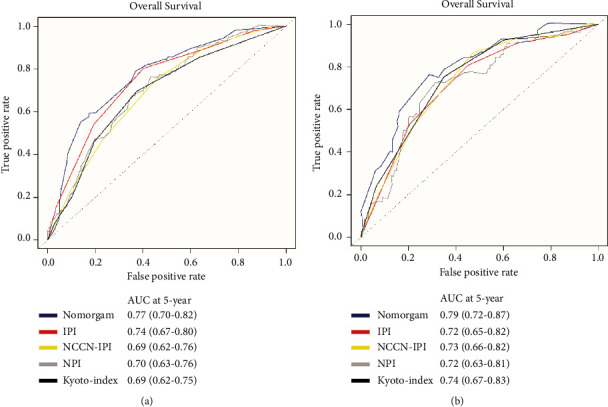
Comparison with current prognostic scoring systems. ROC curves for predicting 5-year OS of our predictive model, IPI, NCCN-IPI, NPI, and Kyoto-index in the training (a) and the validation cohort (b). AUC, area under curve; NPI, the model established by Sun Yat-Sen University Cancer Center (China); Kyoto-index, the model established by Kyoto Prefectural University of Medicine (Japan).

Nomograms showed better accuracy in predicting 5-year survival in cohorts compared to IPI, NCCN-IPI, NPI [12], and Kyoto-index [13] (Figures 4(a) and 4(b)). The AUC of the predictive model (0.77, 95% CI: 0.70–0.82) in the training cohort was higher than that of the IPI (0.74, 95% CI: 0.67–0.80), NCCN-IPI (0.69, 95% CI: 0.62–0.76), NPI (0.70, 95% CI: 0.63–0.76), and Kyoto-index (0.69, 95% CI: 0.62–0.76) (*p*=0.013). Likewise, the AUC of IPI (0.72, 95% CI: 0.65–0.82) and NCCN-IPI (0.73, 95% CI: 0.65–0.82) in the validation cohort was lower than that of the predictive model.

**Figure 5:**
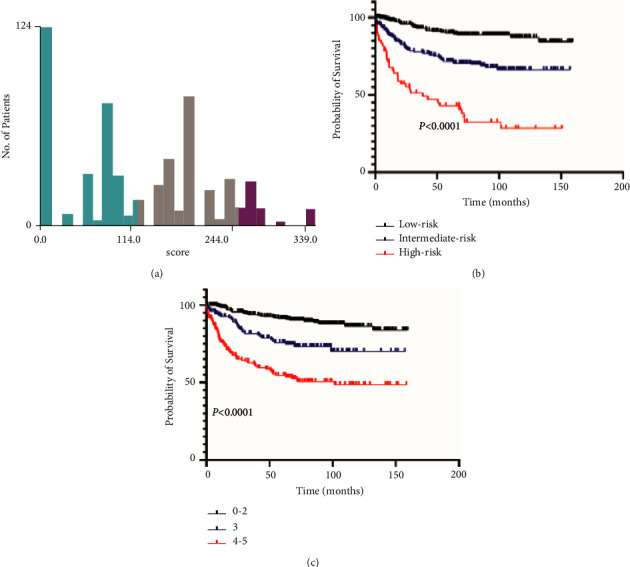
Cutoff values calculated by X-tile (a). Overall survival of patients with diffuse large B-cell lymphoma stratified by risk according to our new model (b) and IPI (c).

We then classified all patients into low-, intermediate-, and high-risk groups based on OS scores generated by nomogram. The cutoff values were determined by X-tile software (Figure 5(a)). The 5-year OS of patients differentiated by International Prognostic Index (IPI) score was 90% (score 0–2), 73% (score 3), and 51% (score 4-5), respectively (Figure 5(c)). The 5-year OS of patients in the low-, intermediate-, and high-risk groups identified by the new predictive model was 89%, 70%, and 33%, respectively (Figure 5(b)).

**Figure 6:**
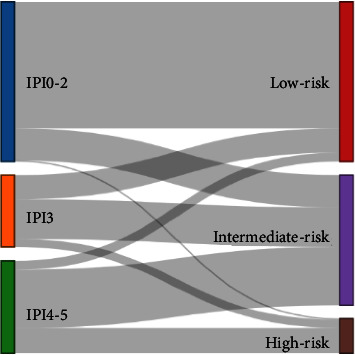
The relationship between the IPI score system and new predictive model shown by Sankey diagram. Panels on the left are grouped according to different IPI scores, and panels on the right are risk subscales based on the new model.

To clearly demonstrate the relationship between IPI scores and the new model's predictions in outcome of DLBCL patients, a Sankey diagram was constructed (Figure 6). We further categorized patients in the high-risk group (point 4-5) defined by the IPI score into subgroups of 117 patients in the nonhigh-risk group and 44 in the high-risk group by the new model. The baseline clinical characteristics of the two subgroups are shown in Table 3.

## 4. Discussion

To our knowledge, our cohort had the best clinical outcome among the reported studies with the same sample size of patients in general hospitals in China.

The median age of patients with DLBCL in our study was 58 years, which was consistent with the data reported by other research centers in Asia [14, 15], but lower than those reported in other continents [16, 17]. Compared with other studies [13, 18–27], especially in cancer hospitals in China [6, 12], our cohort had a higher proportion of patients with advanced stage and combined B symptoms, which indicated that patients in our center have a heavier burden of disease. Primary extranodal DLBCL can originate from almost any part of the body, and the most common site of involvement in our cohort was the gastrointestinal tract. In addition, the involvement of the mammary gland, thyroid gland, and testis also occupied a large portion, which was consistent with previously reported data [28–30].

Multicenter data showed that the 5-year OS of DLBCL is about 64% in the rituximab era [30–32], while the survival of our cohort was better. This may be due to the availability of more new drugs, improvements in supportive care, and appropriate adjustments in treatment regimens. Previous prediction models [2, 4] had shown that age, stage, ECOG PS, bone marrow involvement, and number of extranodal sites are momentous prognostic factors, and the data in our study were consistent. In addition, non-GCB [33] pathological subtype was a predictor of poor prognosis (5-year OS 73%).

Albumin levels are commonly used in lymphoma studies. Decreased albumin levels indicate the poor nutritional status of the patient or the consumption of the tumor. However, studies had shown that low Alb may be driven more by proinflammatory status [34] or increased cytokine release [35] than by nutritional status. Biccler et al. [20] considered Alb as a predictor of poor clinical outcomes for patients with DLBCL. Similarly, our data suggested that patients with low Alb have significantly worse survival than other patients. In addition, McMillan et al. [36] considered albumin levels as a good predictor of disease progression. Patients with low albumin levels, especially older patients, were more prone to coinfection, which was also associated with a worse prognosis [37].

As an easily available biomarker, the role of LMR in predicting the survival of DLBCL had been increasingly emphasized. Absolute monocytes were positively associated with the number of tumor-associated macrophages, while the latter was associated with a worse prognosis of DLBCL [38]. Low absolute lymphocyte count suggested poor immune status and was associated with poor prognosis in patients with DLBCL. Therefore, lower LMR predicted worse clinical outcomes [39]. However, there is no uniform standard for the optimal cutoff value of LMR, and a meta-analysis of patients with DLBCL showed that LMR ranged from 1.6 to 4 [40]. Therefore, the critical point determined by ROC curve in our study is 2.5.

Survival of patients with DLBCL had greatly improved in the last 20 years, but we realized that survival in high-risk patients was still poor. We developed a new prediction model to better distinguish high-risk patients. Based on the original IPI and NCCN-IPI, we removed some variables and added Alb and LMR. After verification of internal and external data, the predictive model we developed proved to have good predictive performance. And this model had better predictive power than those of IPI and NCCN-IPI. High-risk patients differentiated according to our model had a worse prognosis.

The main limitation of our study was that it is a single-center retrospective study and its results may not be fully applicable to all patients with DLBCL. In addition, selection bias was difficult to avoid. The model we developed also needs to be validated by larger samples and external study cohorts.

In summary, we analyzed the clinical features of patients with DLBCL in our center and showed better survival. Then, we constructed a new model with better predictive performance by identifying prognostic risk factors, which may help clinicians to better predict clinical outcomes for patients in the rituximab era.

## Figures and Tables

**Table 1:** Clinical characteristics of patients (*n* = 564).

Characteristics	All patients, *n* (%)	Training cohort, *n* (%)	Validation cohort, *n* (%)	*P* value^a^
Total	564	376	188	
Gender				
Male	282 (50.0)	191 (50.8)	91 (48.4)	0.592
Female	282 (50.0)	185 (49.2)	97 (51.6)	
Age				
≤60	320 (56.7)	208 (55.3)	112 (59.6)	0.336
>60	244 (43.3)	168 (44.7)	76 (40.4)	
COO				
GCB	278 (49.3)	177 (47.1)	101 (53.7)	0.137
Non-GCB	286 (50.7)	199 (52.9)	87 (46.3)	
Ki-67 (%)				
≥90	135 (23.9)	87 (24.8)	48 (26.5)	0.530
<90	429 (76.1)	289 (75.2)	140 (73.5)	
B symptoms				
Presence	289 (51.2)	190 (50.6)	99 (52.7)	0.634
Absence	275 (48.8)	186 (49.4)	89 (47.3)	
ECOG				
0-1	288 (51.1)	190 (50.1)	88 (46.8)	0.810
>1	276 (48.9)	186 (49.9)	90 (53.2)	
Bulky disease				
Presence	77 (13.7)	48 (12.8)	29 (15.4)	0.386
Absence	487 (86.3)	328 (87.2)	159 (84.6)	
Ann Arbor				
I-II	158 (28.0)	103 (27.4)	55 (31.2)	0.643
III-IV	406 (72.0)	273 (72.6)	133 (68.8)	
No. of extranodal sites				
0-1	462 (81.9)	315 (83.8)	155 (87.1)	0.998
>1	102 (18.1)	61 (16.2)	30 (12.9)	
BM involvement				
Presence	71 (12.6)	44 (11.7)	27 (15.2)	0.369
Absence	493 (87.4)	332 (88.3)	161 (84.8)	
CNS involvement				
Presence	36 (6.4)	24 (7.2)	12 (6.8)	1
Absence	528 (93.6)	352 (92.8)	176 (93.2)	
LDH				
Elevated	254 (45.0)	169 (44.9)	85 (48.3)	0.952
Normal	310 (55.0)	207 (55.1)	103 (51.7)	
Alb				
Low	167 (29.6)	109 (30.0)	58 (33.0)	0.648
Normal	397 (70.4)	267 (70.0)	130 (67.0)	
LMR				
Low^b^	223 (39.5)	144 (38.3)	79 (44.9)	0.394
High	341 (60.5)	232 (61.7)	109 (55.1)	
D-dimer				
c > 0.87	332 (58.9)	223 (59.3)	109 (61.9)	0.762
≤0.87	232 (41.1)	153 (40.7)	79 (38.1)	
HBsAg				
Positive	48 (8.5)	34 (9.0)	14 (8.0)	0.522
Negative	516 (91.5)	342 (91.0)	174 (92.0)	
IPI				
0-1	159 (28.2)	108 (28.7)	51 (27.1)	0.928
2	119 (21.1)	80 (21.3)	39 (20.7)	
3	125 (22.2)	84 (22.3)	41 (21.8)	
4-5	161 (28.5)	104 (27.7)	57 (30.3)	

LMR, lymphocyte-monocyte ratio; BM, bone marrow; CNS, central nervous system; COO, cell of origin; ECOG, Eastern Cooperative Oncology Group; ^a^training cohort vs. validation cohort, *P* value was calculated by the Pearson Chi-square test, and *P* < 0.05 is regarded significant. ^b^<2.5.

**Table 2:** Risk factors for survival in patients with DLBCL.

Variables	Univariate, *P* value	Multivariate		
		HR	95%	*P* ^*∗*^ value
Male	0.045			0.151
Age > 60	<0.001	2.086	1.371–3.175	<0.001
ECOG > 1	<0.001	2.666	1.790–3.970	0.001
With B symptom	0.001			0.656
Ann Arbor stage III-IV	<0.001	1.857	1.035–3.333	0.038
IPI > 2	<0.001			
Non-GCB (reference GCB)	0.042			0.393
Ki-67 > 90%	0.251			
Bulky disease	0.148			
BM involvement	<0.001	2.024	1.413–2.898	<0.001
CNS involvement	0.007			0.381
No. of extranodal sites > 1	0.001			0.194
Evaluated LDH	<0.001			0.083
Evaluated D-dimer	0.063			0.873
Low LMR	0.001	1.605	1.128–2.283	0.015
Low ALB	<0.001	1.548	1.088–2.202	0.008
HbsAg positive	0.808			

ECOG, Eastern Cooperative Oncology Group; GCB, germinal center B-cell; CNS, central nervous system; LDH, lactate dehydrogenase; LMR, lymphocyte-monocyte ratio; ^*∗*^*P* value was calculated by Cox regression, and *P* < 0.05 is regarded significant.

**Table 3:** Clinical characteristics of patients with DLBCL (IPI 4-5, *n* = 161).

Characteristics	Nonhigh-risk^a^, *n* (%)	High-risk^a^, *n* (%)	*P* value^b^
Total	117	44	
Gender			
Male	51 (43.6)	20 (45.5)	0.832
Female	66 (56.4)	24 (54.5)	
Age			
≤60	56 (47.9)	6 (13.6)	<0.001
>60	61 (52.1)	38 (86.4)	
COO			
GCB	56 (47.9)	15 (34.1)	0.117
Non-GCB	61 (52.1)	29 (65.9)	
Ki-67 (%)			
≥90	26 (22.2)	9 (20.5)	0.809
<90	91 (77.8)	35 (79.5)	
B symptoms			
Presence	87 (74.4)	38 (86.4)	0.103
Absence	30 (25.6)	6 (13.6)	
ECOG			
0-1	27 (23.1)	0 (0)	<0.001
>1	90 (79.9)	44 (100)	
Bulky disease			
Presence	15 (12.8)	3 (6.8)	0.403
Absence	102 (87.2)	41 (93.2)	
Ann Arbor			
I-II	1 (0.8)	0 (0)	1
III-IV	116 (99.2)	44 (100)	
No. of extranodal sites			
0-1	78 (66.7)	23 (52.3)	0.092
>1	39 (33.3)	21 (47.7)	
BM involvement			
Presence	18(15.4)	25 (56.8)	<0.001
Absence	99 (84.6)	19 (43.2)	
CNS involvement			
Presence	10 (8.5)	8 (18.2)	0.084
Absence	107 (91.5)	36 (91.8)	
LDH			
Elevated	95 (81.2)	36 (81.8)	0.928
Normal	22 (18.8)	8 (18.2)	
Alb			
Low	59 (50.5)	43 (97.7)	<0.001
Normal	58 (49.5)	1 (2.3)	
LMR			
Low^c^	57 (48.7)	35 (79.5)	<0.001
High	60 (51.3)	9 (20.5)	
D-dimer			
>0.87	91 (77.8)	32 (72.7)	0.501
≤0.87	26 (22.2)	12 (27.3)	
HBsAg			
Positive	11 (9.4)	2 (4.5)	0.517
Negative	106 (90.6)	42 (95.5)	

LMR, lymphocyte-monocyte ratio; BM, bone marrow; CNS, central nervous system; COO, cell of origin; ECOG, Eastern Cooperative Oncology Group; ^a^using our new model to distinguish prognostic subgroups. ^b^nonhigh-risk vs. high-risk patients, *P* value was calculated by the Pearson Chi-square test or Fisher's exact test, and *P* < 0.05 is regarded significant. ^c^<2.5.

## Data Availability

The data supporting the findings of this study are included within the article.
